# Comparative analysis of plastid genomes reveals rearrangements, repetitive sequence features, and phylogeny in the Annonaceae

**DOI:** 10.3389/fpls.2024.1351388

**Published:** 2024-04-17

**Authors:** Jingyao Ping, Jing Hao, Ting Wang, Yingjuan Su

**Affiliations:** ^1^ School of Life Sciences, Sun Yat-Sen University, Guangzhou, China; ^2^ College of Life Sciences, South China Agricultural University, Guangzhou, China; ^3^ Research Institute of Sun Yat-Sen University, Shenzhen, China

**Keywords:** Annonaceae, plastomes, phylogeny, IR expansion, repetitive sequences

## Abstract

The Annonaceae stands as the most species rich family in the Magnoliales, a basal group of angiosperms. Widely distributed in tropical and subtropical regions, it holds significant ecological and economic value. The plastid genome (plastome) is often employed in studies related to plant phylogenetics, comparative genomics, evolutionary biology, and genetic engineering. Nonetheless, research progress on plastid genomics in the Annonaceae has been relatively slow. In this study, we analyzed the structure and repetitive sequence features of plastomes from 28 Annonaceae species. Among them, *Mitrephora tomentosa* and *Desmos chinensis* were newly sequenced, with sizes of 160,157 bp and 192,167 bp, and GC contents of 38.3% and 38.4%, respectively. The plastome size in the Annonaceae ranged from 158,837 bp to 202,703 bp, with inverted repeat (IR) region sizes ranging from 64,621 bp to 25,861 bp. Species exhibiting expansion in the IR region showed an increase in plastome size and gene number, frequent boundary changes, different expansion modes (bidirectional or unidirectional), and an increase in repetitive sequences. Specifically, a large number of dispersed repetitive sequences lead to an increase in the size of the LSC region in *Goniothalamus tamirensis*. Phylogenetic analysis revealed Annonoideae and Malmeoideae as monophyletic groups and sister clades, with *Cananga odorata* outside of them, followed by *Anaxagorea javanica*. This research uncovers the structural variation characteristics of plastomes in the Annonaceae, providing valuable information for understanding the phylogeny and plastome evolution of Annonaceae.

## 1 Introduction

Plastids are organelles responsible for photosynthesis in plants and some protists, possessing their own independent genome known as the plastid genome (plastome). They consist of three parts: the large single-copy (LSC) region, the small single-copy (SSC) region, and the inverted repeat (IR) region (Mower and Vickrey, 2018). In recent years, plastomes have been increasingly utilized in phylogenetic evolution studies. Compared to the nuclear genome, plastomes have a smaller genome size, larger copy number, and are easier to obtain. The development of sequencing technologies has greatly facilitated research in plastid genomics, making it easier to obtain complete plastomes and providing more information (Tonti-Filippini et al., 2017). It has been observed that plastome evolution is often accompanied by gene loss or pseudogenization (Chaw et al., 2018), inversion of sequence fragments (Chaw et al., 2018), or expansion and contraction of IR regions (Zhu et al., 2016; Mower and Vickrey, 2018). By comparing features such as genome size, gene content, boundary shift, and structural rearrangement, new insights can be gained for phylogenetic studies and provide rich information for understanding the evolution process of plastomes (Lian et al., 2022). Therefore, studying plastomes is of great significance for revealing phylogenetic relationships between species, conserving and utilizing germplasm resources, as well as improving varieties.

Annonaceae is the largest pantropical family of trees and lianas in the early-divergent order Magnoliales among angiosperms, consisting of 107 genera and approximately 2,300 species. It is divided into four subfamilies: Anaxagoreoideae, Ambavioideae, Malmeoideae, and Annonoideae. Among them, Annonoideae is further divided into 6 tribes: Uvarieae, Monodoreae, Guatterieae, Duguetieae, Bocageeae, Annoneae, and Xylopieae. Malmeoideae is divided into 8 tribes: Dendrokingstonieae, Fenerivieae, Maasieae, Malmeeae, Miliuseae, Monocarpieae, Piptostigmateae, and Phoenicantheae (Chatrou et al., 2012; Guo et al., 2017). The family is widely distributed in tropical and subtropical regions and serves as a significant component of lowland tropical rainforest ecosystems (Punyasena et al., 2008). Moreover, it represents an important source of high-quality fruits, timber, aromatic essential oils, and valuable medicinal materials, possessing both ecological and economic value. In China, the Annonaceae is represented by 24 genera and 120 species, of which 41 species and one variety are endemic to China. It primarily grows in regions south of the Yangtze River (Li and Gilbert, 2011). Additionally, two species are listed as key protected wild plants at the national level (https://www.iplant.cn/rep/protlist). The functional value of Annonaceae varies among genera and species, and a systematic classification study of this family serves as the foundation for functional development. However, the remarkable diversity of Annonaceae has also posed great challenges and controversies in its phylogenetic research. For example, there are controversies regarding the relationships within certain branches and genera, including the *Meiogyne*-*Fitzalania* Clade (Thomas et al., 2012), *Desmopsis*-*Stenanona* Clade (Ortiz-Rodriguez et al., 2018), *Friesodielsia* (Saunders et al., 2020), and *Polyalthia* (Chaowasku et al., 2018; Xue et al., 2021), among others. The development of sequencing technology has greatly advanced research in platid genomics. Recently, Gan et al. (2022) published the plastome of *Miliusa glochidioides* Hand.-Mazz. Ping et al. (2023) published the plastome of the *Trivalvaria costata* (J. D. Hooker and Thomson) I. M. Turner and discovered variations in the IR region among different species of the Annonaceae.

In order to understand and supplement the plastome characteristics of additional Annonaceae species, we have sequenced *Mitrephora tomentosa* Hook. f. & Thomson and *Desmos chinensis* Lour. These two species represent the first within their respective genera to have their plastomes sequenced. *Mitrephora tomentosa* is a deciduous tree with bark that is black to deep gray-black in color and emits a slightly sweet fragrance. This species is distributed in Guangdong, Hainan, and southern Yunnan in China, as well as in Southeast Asia. Its wood is hard and is often utilized for constructing vehicles and buildings. In addition, it is a popular ornamental plant in tropical areas. *Desmos chinensis* is an erect or climbing shrub found predominantly in Guangdong, Guangxi, Yunnan, and Guizhou in China. Its roots and leaves have medicinal properties, and the stem bark fiber can be used as a substitute for cotton and as a raw material for papermaking (Li and Gilbert, 2011). Furthermore, in this study, we additionally selected 26 publicly available plastomes of Annonaceae from the NCBI database (https://www.ncbi.nlm.nih.gov) for the following analyses: 1) Construction of a phylogenetic tree based on concatenated datasets of conserved protein-coding genes. 2) Structural comparisons, including border shifts and variations in the IR regions. 3) Distribution patterns of repetitive sequences.

## 2 Materials and methods

### 2.1 Sampling and sequencing

The fresh leaves of *Mitrephora tomentosa* and *Desmos chinensis* were collected from the South China Botanical Garden, Chinese Academy of Sciences (coordinates: E113°36.9’, N23°18.1’, and E113°36.4’, N23°18.5’), and stored at -80°C. The total DNA was extracted using the E.Z.N. A.^®^ Plant DNA kit (OMEGA, USA), and the DNA was fragmented using a Covaris M220 instrument. After purification, TruSeq™ RNA sample Prep Kit was used to construct the sequencing library. Paired-end sequencing was performed on the Illumina NovaSeq6000 platform with a read length of 150 bp. The raw data were filtered using Cutadapt v1.16 software (Martin, 2011), followed by assembly using GetOrganelle V1.7.7.0 software (Jin et al., 2020) with the seed set as gi|12394|emb|V00171.1| Zea mays chloroplast gene for the large subunit of RUBP (ribulose bisphosphate carboxylase), and the database utilized was embplant_pt. The genome was annotated using PGA (Qu et al., 2019) and Geseq (https://chlorobox.mpimp-golm.mpg.de/geseq.html) web-based programs (Tillich et al., 2017), and the sequence was deposited in the NCBI database (https://www.ncbi.nlm.nih.gov), accession numbers: OQ682535 and OQ687053. The plastome map was drawn online using OGDRAW software (https://chlorobox.mpimp-golm.mpg.de/OGDraw.html) (Greiner et al., 2019).

### 2.2 Sequence data and construction of phylogenetic trees

We downloaded 26 additional plastome sequences from the Annonaceae and two species from the Magnoliaceae (as outgroups) from the NCBI database (https://www.ncbi.nlm.nih.gov). The plastome annotation was corrected using the online website GeSeq. In addition to two newly sequenced species, a total of 28 species from the Annonaceae, covering 21 genera, were selected for analysis. Shared protein-coding genes (PCGs) were extracted by importing the sequences into Geneious Prime 2022.0.1 (Kearse et al., 2012), and a concatenated dataset was constructed after alignment. We utilized MEGA X software (Kumar et al., 2018) to predict the best model (GTR+G+I) and construct neighbor-joining (NJ) trees with 1000 bootstrap replications. Additionally, we constructed maximum-parsimony (MP), maximum-likelihood (ML), and Bayesian-inference (BI) trees using PAUP 4.0 software (Swofford, 2002), RaxML 8.0.20 software (Stamatakis, 2014) with GTRGAMMAI model and 1000 bootstrap replicates, and Mrbayes v3.2.0 software (Huelsenbeck and Ronquist, 2001) with 1,000,000 generations (Nst = 6, rates = invgamma), respectively.

### 2.3 Structural and repeat sequence analysis

The global alignment of plastomes was conducted using the Mauve module in Geneious prime 2022.0.1. We extracted each sequence (with the IRa region removed) and aligned them to have the same starting position (*trnH-GUG*), while maintaining consistent transcriptional orientation at the starting position. Due to the arbitrary orientation of the SSC region in several species, we manually adjusted the SSC region to place *ndhF* at the end of the sequence. The gene information on the boundaries of the junction sites of the plastome was visualized using IRscope (https://irscope.shinyapps.io/irapp/) (Amiryousefi et al., 2018). The gene information within the inverted repeat regions was re-annotated and manually aligned.

The MISA (Microsatellite Identification Tool) (Beier et al., 2017) online website was used to predict simple sequence repeats (SSRs) (https://webblast.ipk-gatersleben.de/misa/), with minimal iterations of ten repeat motifs for mononucleotides, six for dinucleotide repeats, and five for tri-, tetra-, penta- and hexa-nucleotides. When the distance between two SSRs was less than 100 bp, they were considered as compound SSRs. Tandem repeat sequences (TRSs) were identified using the online software Tandem Repeats Finder v4.09 (TRF) (http://tandem.bu.edu/trf/trf.html) (Benson, 1999). Default parameters were used in the advanced module, with match, mismatch, and gap parameters set to 2, 7, and 7, respectively. The minimum alignment score was set to 50, the maximum period size was set to 500, and the maximum tandem repeat array size (in bp, millions) was set to 2. The online tool REPuter (http://bibiserv.techfak.uni-bielefeld.de/reputer) (Kurtz and Schleiermacher, 1999) was used to search for dispersed repeat sequences (DRSs) of forward (F) and palindromic (P) in plastomes. The parameters were set as follows: a Hamming distance of 3, a maximum of 500 computed repeats, and a minimal repeat size of 60. There are more DRSs observed in *A. atemoya*, with a maximum of 600 computational repetitions being set. The statistical analysis of the parameters was performed using IBM SPSS v22.0.

## 3 Results

### 3.1 Structural features of plastomes

**Figure 1:**
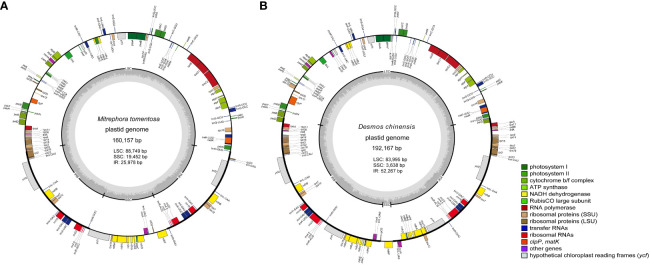
The plastome map of the newly sequenced species. **(A)** *Mitrephora tomentosa*. **(B)** *Desmos chinensis*. Different colored blocks on the genome represent different genes.

The two newly sequenced species, *Mitrephora tomentosa* and *Desmos chinensis*, belong to Malmeoideae and Annonoideae, respectively. The plastome size for *Mitrephora tomentosa* is 160,157 bp, with a GC content of 38.3%, while for *Desmos chinensis*, it is 192,167 bp with a GC content of 38.4%. In *Mitrephora tomentosa*, LSC, SSC, and IR regions have sizes of 88,749 bp (37.6%), 19,452 bp (34.2%), and 25,978 bp (43.3%), respectively, and the whole plastome contains 84 PCGs, 37 tRNA genes, and 8 rRNA genes. Among them, there are 5 PCGs, 7 tRNAs, and 4 rRNA with duplicate copies (**Figure 1A**, **Supplementary Table S1**). In *Desmos chinensis*, the LSC, SSC, and IR regions have sizes of 83,995 bp (37.3%), 3,638 bp (28.5%), and 52,267 bp (39.5%), respectively, and the whole plastome encodes 105 PCGs, 38 tRNA genes, and 8 rRNA genes. Among them, there are 26 PCGs, 8 tRNAs, and 4 rRNA with duplicate copies (**Figure 1B**, **Supplementary Table S1**).

The selected 28 plastomes in the Annonaceae had a size range of 158,837 bp (*Stelechocarpus burahol* (Blume) Hook. f. & Thomson) to 202,703 bp (*Annona atemoya* Mabb.), with a GC content range of 38.4% (*Desmos chinensis*) to 39.9% (*Annona reticulata* L. and *Annona montana* Macf.). The size ranges of LSC, SSC, and IR were 69,649 bp (*Annona squamosa* L.) to 90,803 bp (*Artabotrys hexapetalus* (Linn. f.) Bhandari), 2,948 bp (*Rollinia mucosa* (Jacq.) Baill.) to 20,310 bp (*Cananga odorata* (Lamk.) Hook. f. & Thomson), and 64,621 bp (*Annona reticulata*) to 25,861 bp (*Alphonsea hainanensis* Merr. et Chun), respectively. In addition, the GC content of the LSC (37.3% ~ 38.3%) and SSC (28.5% ~ 35.1%) regions in all species is lower than that of the IR region (39.5% ~ 43.5%). The number of encoded genes ranged from 165 to 129, with PCGs and tRNA genes ranging from 117 to 84 and 40 to 37, respectively, and all species contained eight rRNA genes (**Table 1**). Among them, there are 5 to 37 PCGs and 7 to 10 tRNAs with duplicate copies (**Supplementary Table S1**). At the subfamily level, there is a significant difference in the size distribution of the plastomes among four taxa groups (independent samples Kruskal-Wallis test, *p* < 0.05). In pairwise comparisons, Malmeoideae showed significant differences with Annonoideae (**Supplementary Table S2**).

**Table 1:** Plastid genome information of sampled species.

Species name	NCBI	Genome		LSC		SSC		IR		Gene number			
	Accession number	Size (bp)	GC content (%)	Size (bp)	GC content (%)	Size (bp)	GC content (%)	Size (bp)	GC content (%)	total	CDS	tRNA	rRNA
Annonoideae													
Annoneae													
*Annona atemoya*	MN241495	202,703	39.5	69,685	38.6	2,966	33.6	65,026	40.1	164	116	40	8
*Annona cherimola*	KU563738	201,723	39.6	69,771	38.6	2,966	33.6	64,493	40.2	164	116	40	8
*Annona montana*	MK087989	195,495	39.9	75,172	38.8	3,105	35.1	58,609	40.8	158	112	38	8
*Annona muricata*	MT742546	196,038	39.6	75,339	38.8	3,105	35.1	58,797	40.7	158	112	38	8
*Annona reticulata*	MT742547	219,006	39.9	69,650	38.6	3,014	33.5	64,621	40.2	164	116	40	8
*Annona squamosa*	MN241494	200,335	39.6	69,649	38.6	2,966	33.6	63,860	40.3	164	116	40	8
*Goniothalamus tamirensis*	MN241496	193,002	38.8	87,019	37.7	3,047	32.1	51,468	39.8	150	104	38	8
*Rollinia mucosa*	MN241491	201,666	39.3	70,842	38.5	2,948	33.4	63,938	40	165	117	40	8
Uvarieae													
*Anomianthus dulcis*	MN241490	190,682	38.9	83,727	38	3,715	31.9	51,620	39.9	151	105	38	8
*Desmos chinensis*	OQ687053	192,167	38.4	83,995	37.3	3,638	28.5	52,267	39.5	151	105	38	8
*Fissistigma oldhamii*	MW136266	187,782	38.9	82,584	38	3,074	31.9	51,062	39.8	151	105	38	8
*Fissistigma polyanthum*	MW829282	189,920	38.7	83,000	38	3,273	31.6	51,936	39.5	151	105	38	8
*Monanthotaxis ambrensis*	MN241488	190,122	38.6	83,130	37.6	3,558	30.1	51,717	39.7	151	105	38	8
*Sphaerocoryne affinis*	MN241489	189.597	38.7	83,281	37.7	3,630	29.3	51,343	39.8	151	105	38	8
*Uvaria macrophylla*	MH992130	192,782	38.7	83,581	37.9	3,741	31.5	52,730	39.6	151	105	38	8
Xylopieae													
*Artabotrys hexapetalus*	MZ936420	178,457	38.8	90,803	37.6	3,066	32.2	42,294	40.3	140	94	38	8
*Artabotrys pilosus*	OK216144	178,195	38.8	90,797	37.6	3,098	32	42,150	40.4	140	94	38	8
Malmeoideae													
Miliuseae													
*Alphonsea hainanensis*	MN253543	159,041	39.2	88,771	37.7	18,548	34.4	25,861	43.5	129	84	37	8
*Chieniodendron hainanense*	MK035708	160,497	39.1	89,424	37.6	18,949	34.3	26,062	43.4	129	84	37	8
*Miliusa glochidioides*	OM047203	159,789	39.2	88,782	37.7	18,949	34.4	26,029	43.4	129	84	37	8
*Mitrephora tomentosa*	OQ682535	160,157	38.3	88,749	37.6	19,452	34.2	25,978	43.3	129	84	37	8
*Monoon laui*	OL979152	161,178	39.1	89,555	37.7	18,975	34.3	26,324	43.2	129	84	37	8
*Polyalthia suberosa*	OM937139	159,408	39.2	88,566	37.7	19,000	34.6	25,921	43.5	129	84	37	8
*Polyalthiopsis verrucipes*	MW018366	159,965	39	89,030	37.5	18,987	34.2	25,974	43.4	129	84	37	8
*Stelechocarpus burahol*	MN253544	158,837	39.2	88,218	37.7	18,733	34.4	25,943	43.4	129	84	37	8
*Trivalvaria costata*	OM914484	162,002	39	87,143	37.7	18,817	34.3	28,021	42.8	132	87	37	8
Ambavioideae													
*Cananga odorata*	MN016933	167,946	39	83,620	37.6	20,310	34.4	32,008	42.4	139	94	37	8
Anaxagoreoideae													
*Anaxagorea javanica*	MK087990	174,645	38.7	89,887	37.7	4,354	31.2	40,202	40.3	139	94	37	8
Magnoliaceae(out-group)													
*Liriodendron chinense*	NC_030504	159,429	39.2	87,766	37.8	18,997	34.3	26,333	43.2	129	84	37	8
*Michelia alba*	NC_037005	160,106	39.2	88,137	37.9	18,777	34.2	26,596	43.2	129	84	37	8

### 3.2 Phylogenetic relationship of Annonaceae

**Figure 2:**
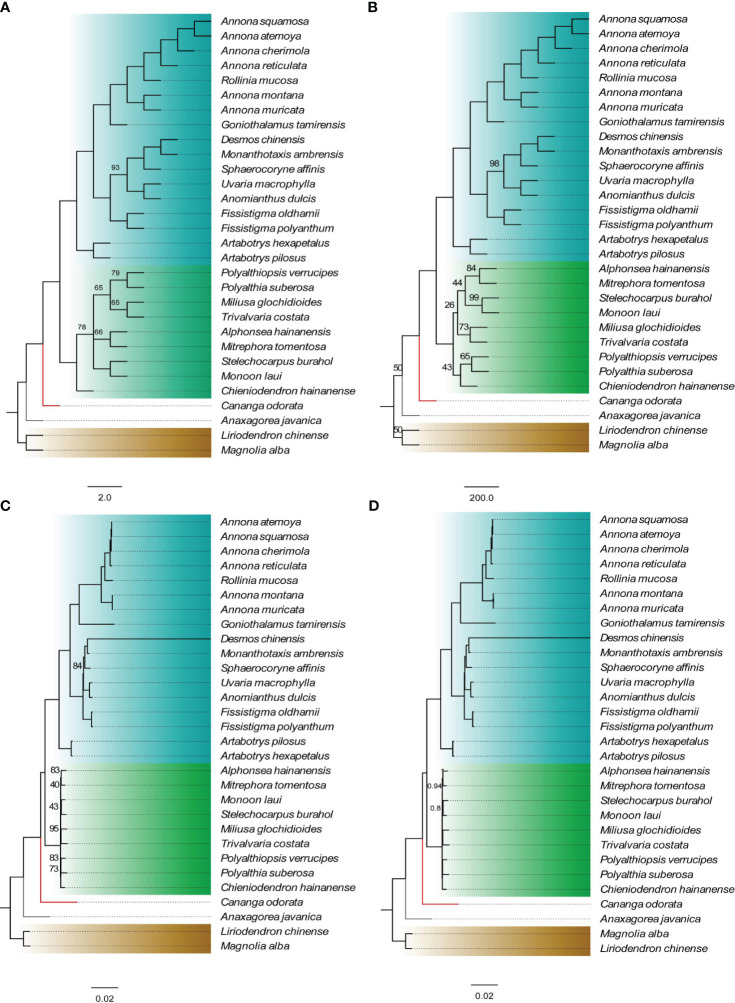
Phylogenetic relationships of Annonaceae based on concatenated data sets of shared protein-coding genes. **(A)** NJ tree. **(B)** MP tree. **(C)** ML tree. **(D)** BI tree. The numbers on branches indicate support values for the respective branches, while the remaining branches have support values of 100 (for **A–C**) or 1 (for **D**). The different colors in the figure represent four subfamilies: blue for Annonoideae, green for Malmeoideae, red branch for Ambavioideae, and gray branch for Anaxagoreoideae.

Using *Michelia alba* DC. and *Liriodendron chinense* (Hemsl.) Sargent. as outgroups, four phylogenetic trees were constructed based on 75 shared PCGs (60,256 bases) and four methods. The results indicate that, except for the Malmeoideae clade in the NJ tree showing a paraphyletic branch, the topologies of the other three trees are completely consistent (**Figure 2**). Annonoideae and Malmeoideae as monophyletic groups and sister clades, with *Cananga odorata* (Ambavioideae) outside of them, followed by *Anaxagorea javanica* Blume (Anaxagoreoideae). Within the subfamily of Annonoideae, the Xylopieae (*Artabotrys hexapetalus* + *Artabotrys pilosus*) are sister to the Annoneae + Uvarieae. The BI tree showed very high branch support, with only two branches having a posterior probability of less than 1. The ML tree had relatively lower branch support, mainly within the Malmeoideae.

### 3.3 Structural rearrangement and boundary shift of plastomes

**Figure 3:**
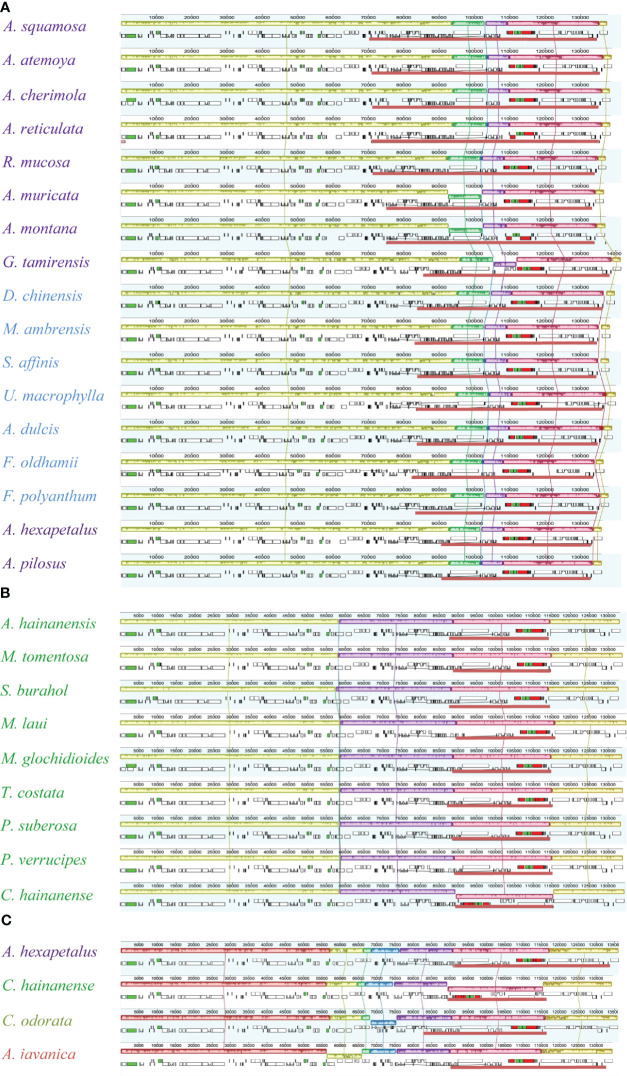
Global alignment of plastomes. **(A)** Alignment of Annonoideae species. **(B)** Alignment of Malmeoideae species. **(C)** Alignment among the four subfamilies.

The Mauve alignment showed that there are 7 collinear blocks in Annonoideae. In comparison to other species, an inversion occurred between *trnV-GAC* and *ndhB* (about 5.9 kb) in *Goniothalamus tamirensis* Pierre ex Finet & Gagnep., while another inversion was identified in the *trnI-CAU*-*ycf2*-*trnL-CAA* (about 9 kb) region in *Annona montana* and *Annona muricata* L. In Malmeoideae, there are 5 collinear blocks, and the entire IR region of *Chieniodendron hainanense* (Merr.) Tsiang et P. T. Li is reversed compared to other species. There are 7 collinear blocks among representative species from the four subfamilies (**Figure 3C**). In *Cananga odorata*, the *psbJ* ~ *5’-rps12* (about 6 kb) fragment underwent inversion, while the *ycf4* ~ *atpE* (8.5 kb) fragment underwent inversion in *Anaxagorea javanica*.

**Figure 4:**
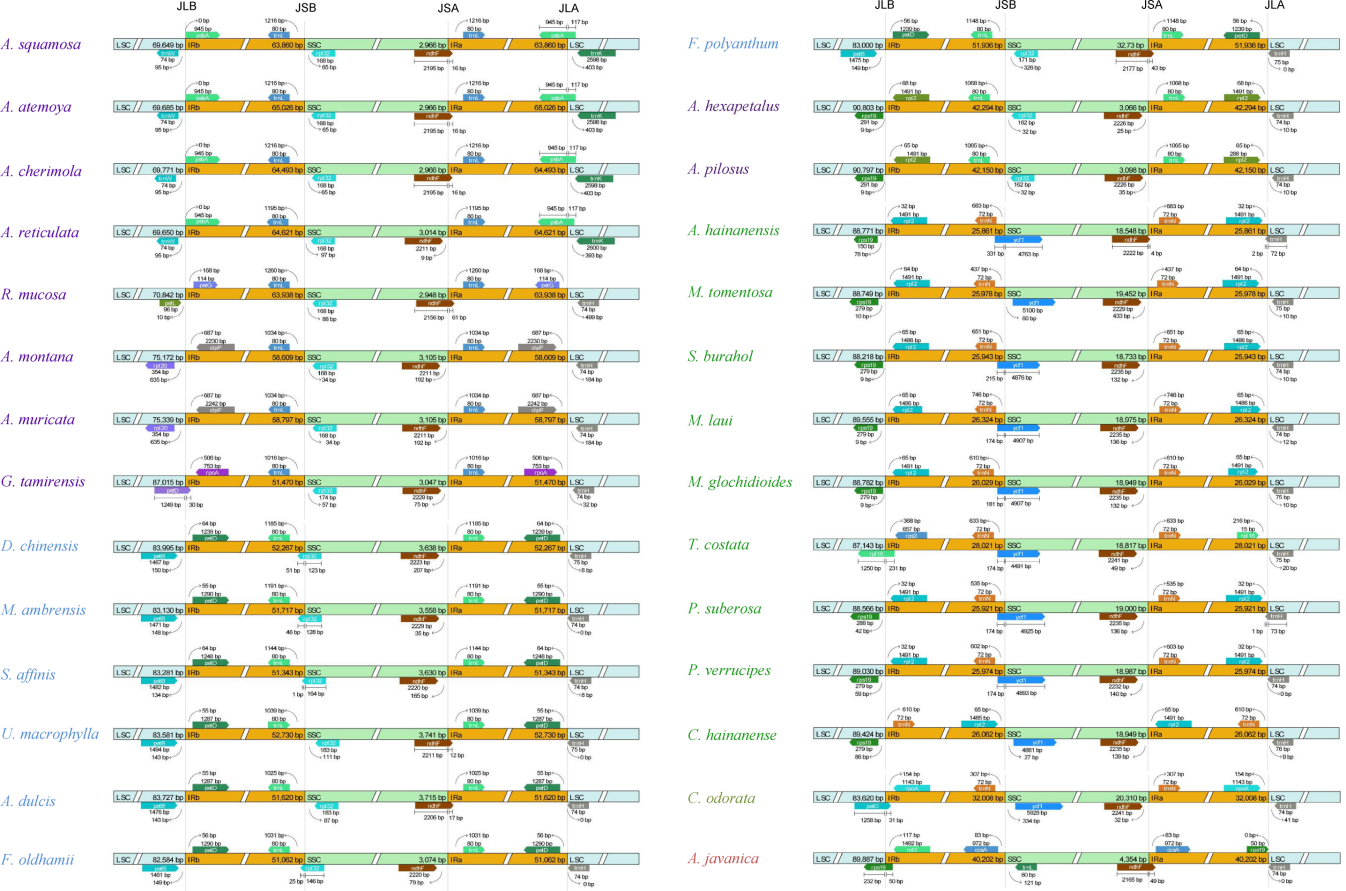
Comparison of the border positions of LSC, SSC, and IR regions among the plastomes of Annonaceae species.

The genes located at the boundaries between the IR and SC regions vary with the changes (expansion) of the IR region (**Figure 4**). In the Annonoideae, the IR region has expanded to include all genes typically found within the SSC except *rpl32* and *ndhF*. In *Annona squamosa*, *Annona atemoya*, *Annona cherimola* Mill., *Rollinia mucosa*, *Uvaria macrophylla* Roxb., *Anomianthus dulcis* (Dunal) J. Sinclair, and *Fissistigma polyanthum* (Hook. f. et Thoms.) Merr., a partial sequence of *ndhF* is located in the IR region. In *Desmos chinensis*, *Monanthotaxis ambrensis* (Cavaco & Keraudren) Verdc., *Sphaerocoryne affinis* (Boerl.) Ridl., and *Fissistigma oldhamii* (Hemsl.) Merr., a partial sequence of *rpl32* is located in the IR region. In Malmeoideae, the genes adjacent to the boundary between the SSC and IR regions are *ndhF* and *ycf1*, with consistent orientation, and *trnH* is located at the LSC/IRa boundary. Except for *Chieniodendron hainanense* and *Mitrephora tomentosa*, the *ycf1* of other species all spans the IRb/SSC boundary. The gene types at the boundary between the IR region and the SSC region in *Chieniodendron hainanense* is opposite to those in other Malmeoideae species (except *Trivalvaria costata*). In *Trivalvaria costata*, *rps16* spans the LSC/IRb boundary. In *Cananga odorata* (Ambavioideae), the LSC/IRb boundary is the same as in *Goniothalamus tamirensis*, where a partial sequence of the *petD* is located at the IR region. The neighboring genes at the boundary between the SSC and IR regions are the same as in Malmeoideae, which are *ndhF* and *ycf1*. However, *ycf1* is entirely located at the SSC region. In *Anaxagorea javanica* (Anaxagoreoideae), *rps19* and *ndhF* span the LSC/IRb boundary and SSC/IRa boundary, respectively. *trnL* and *ccsA* are located at the SSC/IRa boundary and IR/SSC boundary, respectively.

### 3.4 Different expansion patterns of IR regions

**Figure 5:**
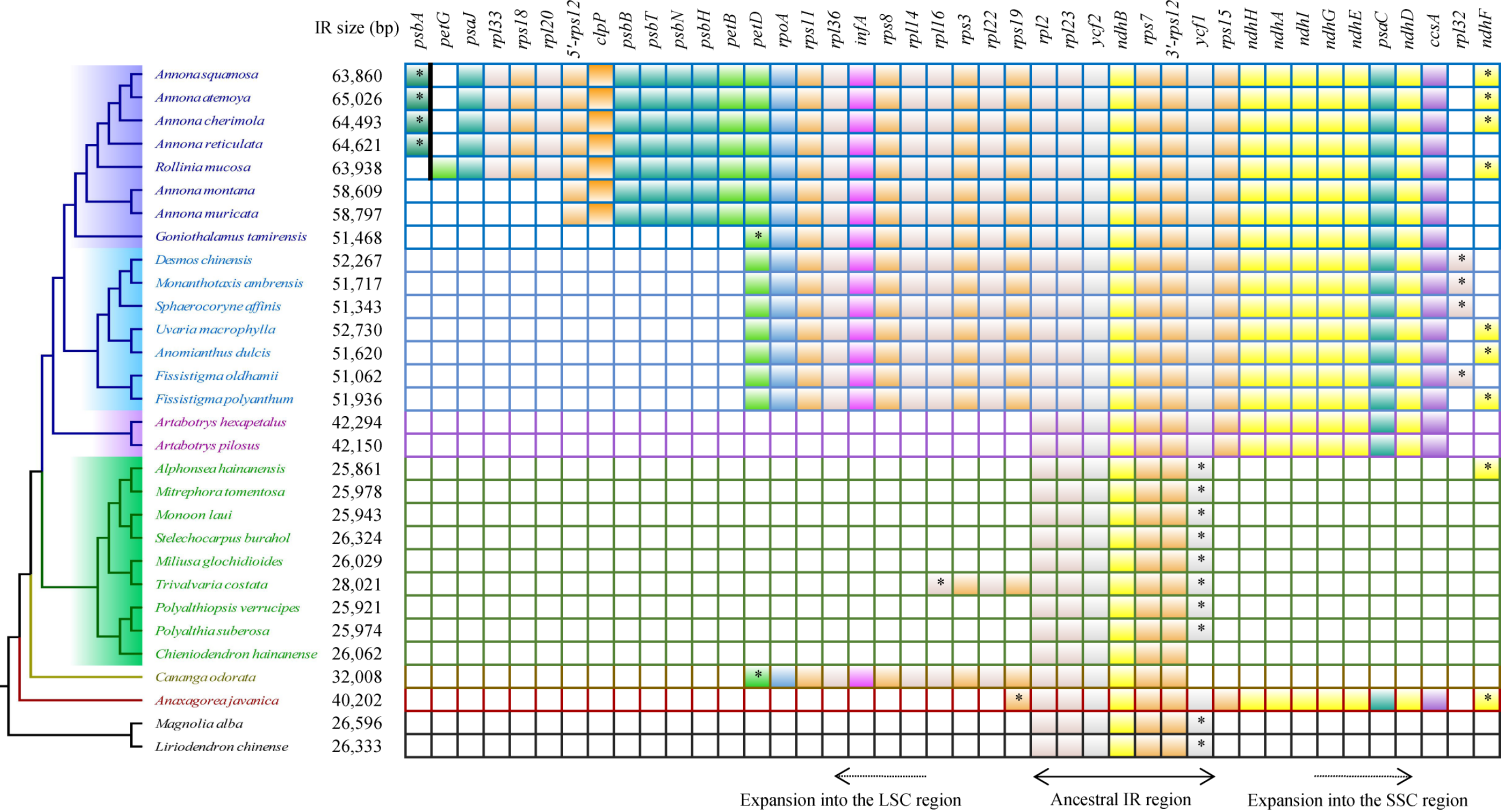
Protein-coding genes that enter the IR region in different species. The “*” represents the part of the gene that enters the IR region. The *psbA* and *petG* are separated by a bold black line to indicate that they are not adjacent.

Compared to the outgroups, 20 species within the Annonaceae displayed varying degrees of expansion in their IR regions. These species include Annonoideae, *Trivalvaria costata*, *Cananga odorata*, and *Anaxagorea javanica*. Within the Annonoideae, the expansion of the IR region was bidirectional, occurring in both the LSC and SSC regions, except for *Artabotrys*. The IR region incorporated a significant portion of the SSC region, approximately 15 kb, which included 10 PCGs: *ycf1*, *rps15*, *ndhH*, *ndhA*, *ndhI*, *ndhG*, *ndhE*, *psaC*, *ndhD*, and *ccsA*. The extent of IR expansion into the LSC region varied among different species. In *Annona atemoya*, *Annona squamosa*, *Annona cherimola*, and *Annona reticulata*, 23 (or 22) PCGs are located at the IR region, totaling about 20 kb. For *Annona muricata* and *Annona montana*, 18 PCGs entered the IR region, totaling about 13 kb. The LSC regions of *Goniothalamus tamirensis* and Uvarieae each had 11 PCGs entering the IR region, totaling about 5 kb. In the Annonoideae, only sequences containing the *ndhF* and *rpl32* were retained in the SSC region, totaling about 3 kb. In contrast, the expansion of the IR region was unidirectional in these five species. *Artabotrys* and *Anaxagorea javanica* incorporated the majority of sequences from the SSC region, totaling approximately 15 kb. In *Trivalvaria costata* and *Cananga odorata*, the IR region expanded into the LSC region by approximately 2 kb and 5 kb, respectively (**Table 1**, **Figure 5**; **Supplementary Table S1**).

For the number and types of tRNA genes located in the IR region of each species (**Supplementary Table S1**), seven tRNA genes (*trnI-CAU*, *trnL-CAA*, *trnV-GAC*, *trnI-GAU*, *trnA-UGC*, *trnR-ACG*, and *trnN-GUU*) are present in all sampled species. *trnL-UAG* is found in Annonoideae; *trnP-UGG* and *trnH-GUG* exist in *Annona squamosa*, *Annona atemoya*, *Annona cherimola*, and *Annona reticulata*; *trnP-UGG* and *trnW-CCA* are present in *Rollinia mucosa*.

### 3.5 The distribution patern of SSRs in the plastome

**Figure 6:**
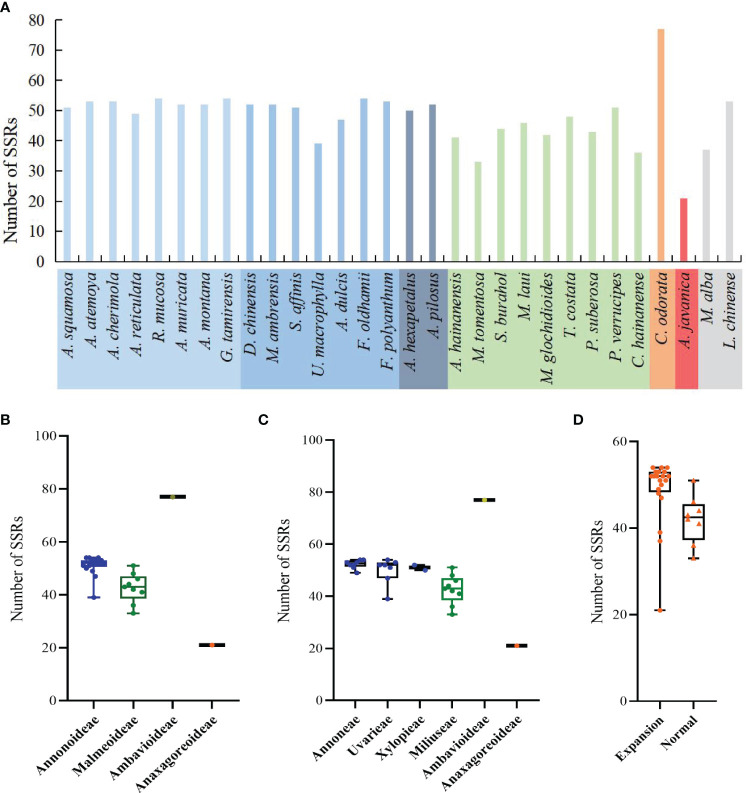
Distribution of SSRs in the plastomes of the Annonaceae. **(A)** Number per species. **(B)** Distribution among the four subfamilies. **(C)** Distribution among the six tribes. **(D)** Distribution between species with IR expansion and other species.

In the sample species of Annonaceae, a total of 1350 SSRs were detected, including 6 nucleotide types and 19 motif types. *Anaxagorea javanica* had the fewest SSRs with 21, while *Cananga odorata* had the most with 77 (**Figure 6A**). The number of mononucleotide SSRs was the highest with 1207 (89.4%), and tetranucleotide SSRs were the least, only found in four species and accounting for 0.5% (**Supplementary Table S3**). A/T was the dominant mononucleotide SSR motif, accounting for 97.6%. The hexanucleotide SSR motif was the most diverse, with six different types (**Supplementary Table S4**). Each species had mononucleotide SSRs as its main type, accounting for 100% (*Polyalthia suberosa*) to 55.6% (*G. tamirensis*). Rank-sum test results show that there are significant differences in the distribution of SSRs among different taxa, including at the subfamily level (**Figure 6B**), tribe level (**Figure 6C**), and between IR expansion and regular sequences (**Figure 6D**). The paired comparison results (**Supplementary Table S5**) indicate significant differences between Ambavioideae and Anaxagoreoideae (*P* = 0.02), Annonoideae and Malmeoideae (*P* = 0.001), as well as Miliuseae and Annoneae (*P* = 0.01).

**Figure 7:**
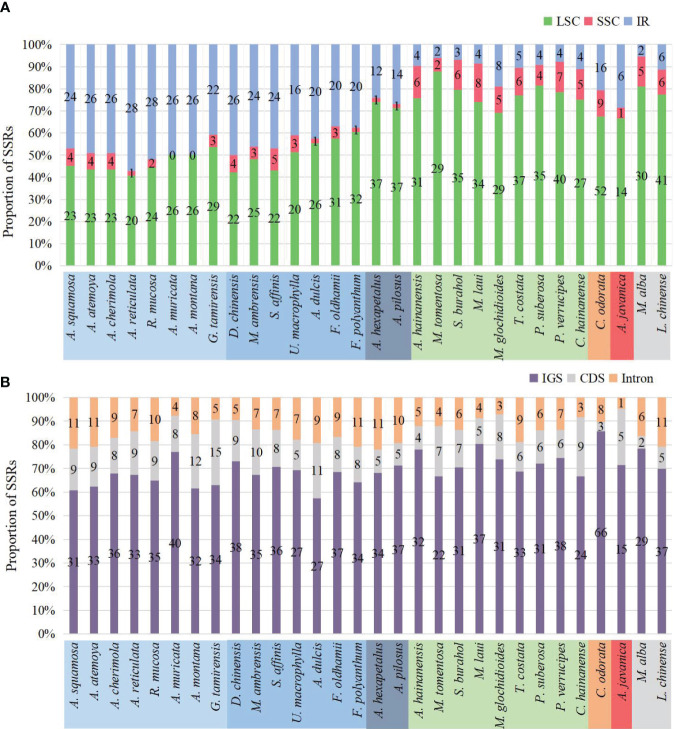
Distribution of SSRs on the plastome. **(A)** Distribution of SSRs in LSC, SSC, and IR. In species where IR has not expanded, SSRs are mainly located in LSC. In species where IR has expanded, the number of SSRs located in the IR region increases. **(B)** Distribution of SSRs in IGS, CDS, and introns. The SSRs of all species are mainly located in the IGS region.

The number of SSRs distributed in the LSC, SSC, and IR regions varied among species. In species without IR expansion, the majority of SSRs were located in the LSC region (88% to 74%). In species with IR expansion, there was a significant increase in SSRs in the IR region, some of which exceeded those in the LSC region, and the number of SSRs located in the SSC region was the least (0 to 5) (**Figure 7A**).

The number of SSRs distributed in different sequence types also varied. Across all species, they were mainly located in the intergenic spacer (IGS) region, ranging from 15 in *Anaxagorea javanica* to 66 in *Cananga odorata*, accounting for 57.4% in *Annona dulcis* to 85.7% in *Cananga odorata*. Compared to the IGS region, there were fewer SSRs located in coding sequence (CDS) and introns (**Figure 7B**). The genes in the coding sequences where SSRs were detected included: *rpoA*, *rpoB*, *rpoC2*, *rpl20*, *rps18*, *rps19*, *ycf1*, *ycf2*, *clpP*, *accD*, *infA*, and *psbF*. The genes containing SSRs in introns include: *atpF*, *clpP*, *trnG-UCC*, *rpl16*, *rps16*, *ndhA*, *ycf3*, *trnK-UUU*, *trnV-UAC*, and *trnL-UAA* (**Supplementary Table S4**).

### 3.6 The distribution of tandem repeat sequences and dispersed repeat sequences in the plastome

**Figure 8:**
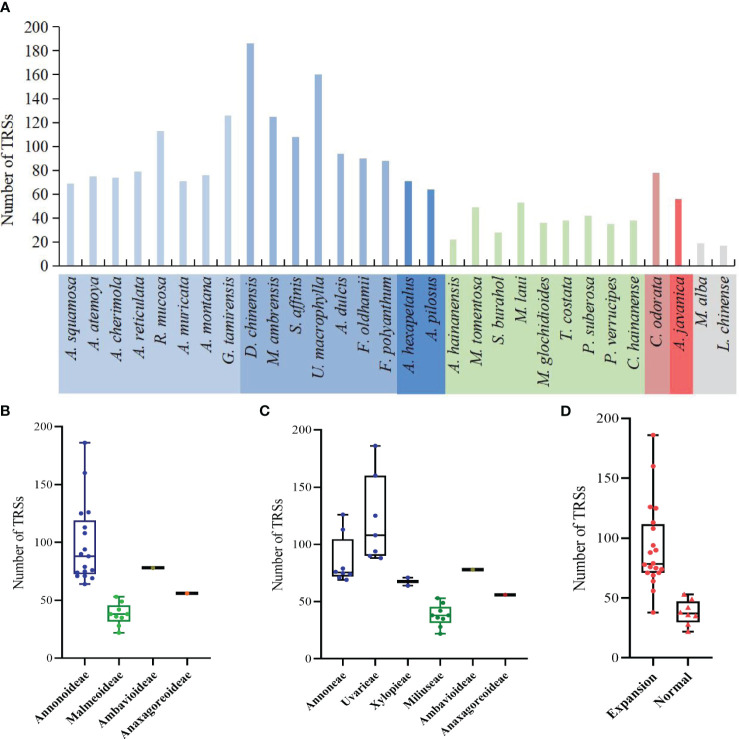
Distribution of the number of tandem repeat sequences in species. **(A)** Number per species. **(B)** Distribution among the four subfamilies. **(C)** Distribution among the six tribes. **(D)** Distribution between species with IR expansion and other species. Rank-sum test results show significant differences between different subfamilies in **(B)**, between different tribes in **(C)**, and between species with IR expansion and other species in **(D)**.

The study detected a total of 2144 TRSs, with the fewest located in the SSC region, followed by the LSC region (**Supplementary Table S6**). The range of TRSs in the Annonaceae varies from 22 (*Alphonsea hainanensis*) to 186 (*Desmos chinensis*) (**Figure 8A**). Among the subfamilies, Annonoideae has the highest number of TRSs, ranging from 64 to 186, and is mainly located in the IR region. Malmeoideae has fewer TRSs, with a range of 22 to 53 (**Figure 8B**). At the tribal level, the Uvarieae species have a relatively higher number of TRSs, ranging from 88 to 186 (**Figure 8C**). The pairwise comparison results indicate significant differences between Annonoideae and Uvarieae, as well as significant differences between Miliuseae and both Annoneae and Uvarieae (**Supplementary Table S5**). A higher number of TRSs was detected in species with expanded IR regions (**Figure 8D**), and the rank-sum test results revealed significant differences in the quantity of TRSs between species with expanded IR regions and other species.

**Figure 9:**
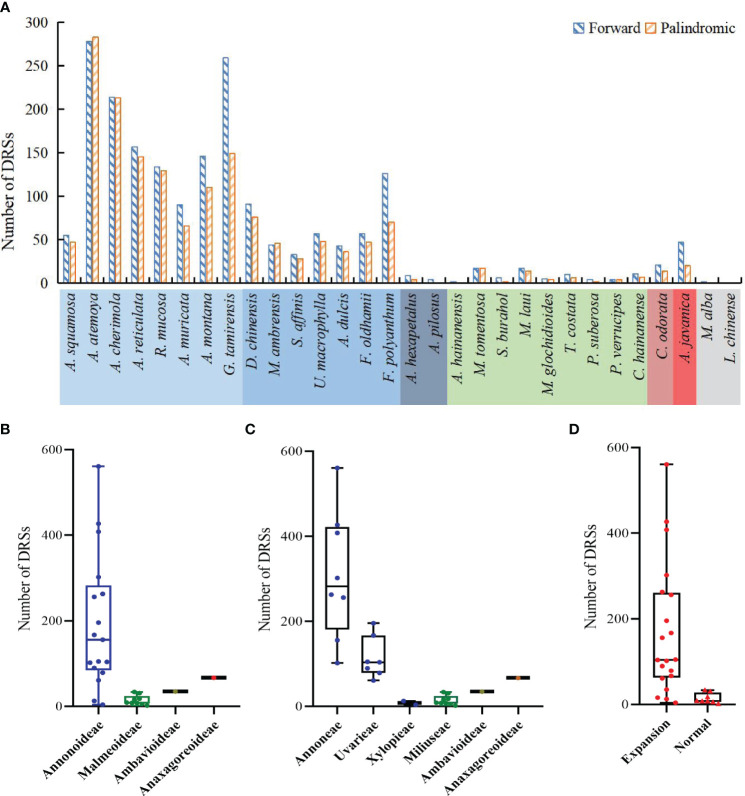
Distribution of dispersed repeat sequences in sampled species. **(A)** Number per species. **(B)** Distribution among the four subfamilies. **(C)** Distribution among the six tribes. **(D)** Distribution between species with IR expansion and other species. Rank-sum test results show significant differences in the number of dispersed repeat sequences among different subfamilies in **(B)**, among different tribes in **(C)**, and between species with IR expansion and other species in **(D)**.

The study detected both forward DRSs (F-DRSs) and palindromic DRSs (P-DRSs), totaling 3,528 in the Annonaceae (**Supplementary Table S7**). *Annona atemoya* was found to have 278 F-DRSs and 283 P-DRSs. Only 2 F-DRSs were detected in *Alphonsea hainanensis* (**Figure 9A**). At the subfamily level, in Annonoideae, except for *Annona squamosa*, *Sphaerocoryne*, and *Artabotrys*, the number of DRSs in the remaining species ranges from 79 to 500 (**Figure 9B**). At the tribal level, Annoneae has the highest number, ranging from 102 to 561, followed by Uvarieae (**Figure 9C**). Most species with expanded IR regions contain a higher number of DRSs (**Figure 9D**). Rank-sum test results show that there are significant differences among different taxa, including at the subfamily level, tribe level, and between IR expansion and regular. The pairwise comparison results (**Supplementary Table S5**) indicate significant differences between Annonoideae and Malmeoideae (*P* = 0.003), Annoneae and Xylopieae (*P* = 0), and as well as Miliuseae and Uvarieae (*P* = 0).

We noticed that *Goniothalamus tamirensis* showed a comparable level of expansion in the IR region as Uvarieae (**Figure 5**). However, the size of the LSC region in *Goniothalamus tamirensis* (87,019 bp) was approximately 4 kb larger than that of Uvarieae (82,584 bp ~ 83,995 bp) (**Table 1**). Through an analysis of repetitive sequence distribution, we discovered 114 DRSs (about 2 kb) in the LSC region of *Goniothalamus tamirensis*, with 113 of them being F-DRSs. In contrast, Uvarieae species had 4 to 24 DRSs in their LSC region (**Supplementary Table S7**). Additionally, *Cananga odorata* also showed a similar extent of expansion from the IR region to the LSC (**Figure 5**), and its LSC size (83,620 bp) was comparable to that of Uvarieae (**Table 1**). Only three F-DRSs were detected in the LSC region of *Cananga odorata* (**Supplementary Table S7**). Therefore, the presence of a significant number of F-DRSs is the primary contributing factor to the enlargement of the LSC region in *G. tamirensis*. In addition, *Goniothalamus tamirensis* has more TRSs (126) than other Annoneae species, especially in the LSC region (58, **Supplementary Table S4**).

## 4 Discussion

### 4.1 The expansion of the IR region drives the enlargement of the plastome in Annonaceae

The size of most land plant plastomes is typically between 120 and 160 kb. We have observed a gradual enlargement of plastomes in Annonaceae, ranging from 159 kb to 201.9 kb. The plastome size of Annonoideae exceeds 178 kb, and the size of the IR region falls between 42 kb and 64 kb. This exceeds the typical size range of the IR region in most land plants, which is 15 kb to 30 kb (Zhu et al., 2016). Among the land plants with sequenced plastomes, it is not common to find plastomes larger than 200 kb. According to incomplete statistics in the NCBI database, there are approximately 32 land plant species with plastomes larger than 200 kb. This includes six *Pelargonium* species (Geraniaceae), 17 *Rhododendron* species (Ericaceae), two *Cypripedium* species (Orchidaceae), *Vitis vinifera* L. (Vitaceae), *Magnolia stellata* (Siebold et Zucc.) Maxim. (Magnoliaceae), and five *Annona* species in this study (**Supplementary Table S8**). The sizes of these genomes range from 200,001 bp to 242,575 bp, with GC content ranging from 28.2% to 39.9%.

The expansion of plastomes is mainly caused by the duplication of genes within the IR region (Sinn et al., 2018; Li et al., 2020), or the expansion of non-coding repetitive sequences (Dugas et al., 2015; Li et al., 2020). The largest plastome in angiosperms is observed in *Pelargonium transvaalense* R.Knuth (242,575 bp), where the IR region expands to 87,724 bp (Weng et al., 2017). The expansion of *Pelargonium* plastomes involves the movement of the IR region into the SSC region, causing the majority of the SSC region to enter the IR region and resulting in the replication of numerous gene fragments (Chumley et al., 2006; Weng et al., 2017). Guo et al. (2021) found that in *Cypripedium*, the expansion occurs in the LSC region, while the lengths of the IR and SSC regions remain unchanged, and the expansion is closely related to the increase of non-coding sequences. Repetitive sequences also play a significant role in genome enlargement. Zhang et al. (2022) observed that the enlarged plastomes in *Cypripedium* have more long sequence repeats and SSRs, suggesting that the increase in repetitive sequences contributes to the obvious enlargement of the genome. Li et al. (2020) proposed that the expansion of *Magnolia liliflora* Desr. plastome is mainly associated with the significant expansion of the IR region and the presence of abundant repetitive sequences. In this study, the enlargement of Annonoideae plastomes is primarily attributed to the expansion of the IR region, with a single IR region expanding by approximately 16 kb to 38 kb. Similarly, in the Annonaceae, the expansion of the IR region directly leads to the enlargement of the plastome.

The GC content also changes with the expansion of the genome. Guo et al. (2021) observed that the plastome of *Cypripedium subtropicum* S. C. Chen & K. Y. Lang (212,668 bp) had a lower GC content (28.8%), which may be related to the increase of AT-biased non-coding regions. Kim et al. (2015) also suggested that the high AT content may be caused by repetitive sequences composed of A and T in the non-coding regions of the single-copy region. Unlike the decrease in GC content, Among these five *Annona* species, the GC content of the plastomes ranged from 39.3% to 39.6%, significantly higher than other Annonaceae species (*P* = 0.002). We observed that the GC content of the LSC region was also significantly higher than other Annonaceae species, which may influence the increase in GC content of the plastome. In addition, consistent with observations in other taxa (Hishamuddin et al., 2020; Zhang et al., 2022), the GC content of the LSC and SSC regions of Annonaceae plastomes was significantly lower than that of the IR region, which is associated with a decrease in A/T bases in the four rRNA genes within the IR region.

### 4.2 Structural rearrangements of plastomes in Annonaceae

In some taxa, the plastome undergoes structural changes during the process of evolution, typically involving rearrangements, expansion of the IR regions, and an increase in sequence repetitions. In the Ericaceae, for example, there are over 20 species with plastomes larger than 200 kb, and within this family, there are rearrangements, expansion of the IR regions, variations in genome size, and repetition of fragments (Li et al., 2020). In the Annonaceae, through global alignment (**Figure 3**) and boundary analysis (**Figure 4**), we identified four inversion events (**Figure 3**), including *trnV-GAC* ~ *ndhB* in *Goniothalamus tamirensis*, *trnI-VAU*-*ycf2*-*trnL-CAA* in *Annona montana* and *Annona muricata*, *psbJ* ~ *5’-rps12* in *Cananga odorata*, and *ycf4* ~ *atpE* in *Anaxagorea javanica*. The occurrence of different inversions in different species suggests that these events may have happened independently. Inversions in the entire IR region are relatively rare, appearing only in *Chieniodendron hainanense* within the Annonaceae. We cannot confirm the existence of this scenario and further sampling is required for verification. Inversions in plastomes are a common occurrence, and some events are used as evidence for classification. For instance, Raubeson and Jansen (1992) discovered that the presence of an approximately 35 kb inversion in the LSC region of lycophytes constitutes strong evidence for their differentiation from angiosperms.

The expansion of the IR region in the Annonaceae is dynamic. In Annonoideae (except for *Artabotrys*), the IR region expanded into both the SSC and LSC regions. In Malmeoideae, only *Trivalvaria costata* was found to have an expansion of its IR region towards the LSC region by 2 kb. Based on the dynamic changes observed in the IR region from the available data, we speculate that the IR region in some other species within Malmeoideae may have also undergone expansion. In the basal group, *Anaxagorea javanica* primarily expanded its IR region towards the SSC region, while in *Cananga odorata*, the IR region mainly expanded towards the LSC region. In addition, the expansion of IR region into SSC region in Annonoideae includes *trnL-UAG*, while it is absent from the IR region of *Anaxagorea javanica* (**Figure 4**). The distinct expansion patterns suggest that they may have independent origins. Large expansions of IR regions have been observed in multiple lineages (Chumley et al., 2006; Weng et al., 2017; Sinn et al., 2018). Some studies suggested that IR region expansion was related to the Poly(A) tracts. Goulding et al. (1996) found a 12 kb expansion of the IR region in the plastomes of *Nicotiana acuminata* (Graham) Hook. The junction between the IR and LSC was located in the intron 1 of *clpP*, and they proposed that this expansion occurred through double-strand DNA breaks and recombination between the poly(A) tracts of *clpP* intron 1 and upstream of *rps19*. The expansion of the IR in *Inga* is located between *ndhD* and SSC, with this region being rich in 78% AT content and containing many potential poly(A) tracts (Dugas et al., 2015). Unlike the massive expansion of IR regions, IR regions are sometimes lost (Ruhlman et al., 2017; Cauz-Santos et al., 2020) or contracted (He et al., 2020) in the plastomes of some plant lineages and algae. Previous studies have shown that many characteristics in Annonaceae are closely associated with its high diversification rate, such as climbing habits, day-night rhythm of pollen adhesion, monoecy, and seed dispersal with seeds enclosed in single-seeded fruits (Xue et al., 2019). We observed three different arrangement patterns in the plastomes of seven species within the *Annona*, demonstrating the diversity of plastome structures within this genus. Overall, significant structural variations exist in the plastomes of the Annonaceae. Some structural features, particularly the varying degrees of IR expansion, can be utilized for phylogenetic research.

Due to the large expansion of the IR region, there was a large variation in the gene number of Annonaceae (129-165). In Annonoideae, 10 to 32 protein-coding genes enter the IR region in Annonoideae, which directly increases the total number of genes. Notably, in *Annona*, 5’-*rps12* is located in the IR region, which is rarely reported before (Ping et al., 2023). In ferns and gymnosperms, it has been found that 3’-*rps12* enters or leaves the IR region as the IR region expands or contracts, and it has been shown that 3’-*rps12* in the IR region has reduced substitution rates and a more conserved sequence signature (Ping et al., 2021b, c). The number of genes was greatly influenced by changes in the IR region, but the types of PCGs were almost identical.

### 4.3 Phylogenetic relationship of Annonaceae

Due to the extensive diversity of Annonaceae, phylogenetic studies on this family have always been subject to controversy (Maas et al., 2015; Chatrou et al., 2018; Ortiz-Rodriguez et al., 2018). In recent years, molecular evidence, primarily derived from plastome data, has successfully aided in resolving some ambiguous classification issues. Tang et al. (2015) utilized sequences from nine plastomes to demonstrate the monophyly of *Goniothalamus*. Xue et al. (2020) constructed a phylogenetic tree using chloroplast genes (*matK*, *rbcL*, and *trnL-F*) and provided evidence that *Polyalthia amoena* A. C. Sm., *Polyalthia capillata* A. C. Sm., and *Polyalthia loriformis* Gillespie belong to *Huberantha*. Wang et al. (2021), through phylogenetic analysis of five chloroplast regions (*psbA-trnH*, *trnL-F*, *matK*, *rbcL*, and *atpB-rbcL*), explicitly placed *Meiogyne kwangtungensis* P. T. Li within the branch of *Pseuduvaria*. Ping et al. (2023) constructed a phylogenetic tree of 65 magnoliid species using shared PCGs and found support for Annonoideae and Malmeoideae as sister groups. In our study, we investigated the phylogenetic relationships of 28 Annonaceae species based on 75 shared plastid PCGs. Our results support Annonoideae and Malmeoideae as monophyletic groups and sister clades, with *Cananga odorata* outside of them, followed by *Anaxagorea javanica*. Additionally, we observed that the phylogenetic relationships within Malmeoideae species had low support in all trees except the BI tree, consistent with previous research (Ping et al., 2023). Furthermore, we found that *Chieniodendron hainanense* is located within Malmeoideae, which is consistent with the findings of Thomas et al. (2012), who placed *Chieniodendron* within *Meiogyne* (Malmeoideae). Considering the various expansion patterns of the IRs within the Annonaceae, we believe that these structural changes can serve as a basis for systematic classification. However, the number of reported plastomes in Annonaceae is currently limited, necessitating the addition of more data that can be combined with morphological features to elucidate the relationships and evolutionary processes within Annonaceae.

### 4.4 Repetitive sequences contribute to the enlargement of plastome size

Repetitive elements play an important role in the structure and size stability of plastomes (Wu et al., 2021). Recently, Li et al. (2023) found a significant correlation between the number of repetitions and tandem repeats with the size of the IR region and plastome in Alismatidae. Zhou et al. (2022) discovered that short repeats and intermediate repeat regions in *Selaginella* mediate various conformations, resulting in diverse and complex structures in plastomes, and identified six new configurations. Long repetitive sequences play an important role in genome rearrangement and are often used to study phylogenetic relationships between species; furthermore, they promote intermolecular recombination in plastomes to generate diversity (Park et al., 2017). This study found that species with expanded IR regions, particularly in Annonoideae, tend to have more repetitive sequences. And the expansion of the LSC region in *Goniothalamus tamirensis* is mainly due to the presence of a large number of forward dispersed repetitive sequences, indicating that forward dispersed repetitive sequences play an important role in plastome size.

SSRs, as molecular markers, are commonly used for studies on genetic diversity, population structure, genetic mapping, phylogenetics, and variety identification (Potter et al., 2015; He et al., 2020). Similar to most species (Wu et al., 2021; Li et al., 2023), SSRs in Annonaceae are mainly composed of mononucleotides and A/T bases. However, Liu et al. (2021) found that SSRs in Polypodiaceae are mainly composed of G/C mononucleotides and predominantly located in the LSC region, consistent with the distribution of SSRs in Malmeoideae. Due to the expansion of the IR region in Annonoideae, the number of SSRs in the LSC and IR are close to each other. SSRs in Annonaceae are mainly located in the intergenic spacer region (IGS), similar to observations in ferns (Liu et al., 2021; Zhu et al., 2021) and cypresses (Ping et al., 2021a). Additionally, Zhu et al. (2021) found that the distribution of SSRs in Cyatheaceae shows intergeneric specificity, providing information for phylogenetic studies within the family.

## 5 Conclusion

Within Magnoliales, the Annonaceae is the most diverse at both the genus and species levels, and existing data demonstrate the diversity of plastomes within this family, including genome expansion, varying degrees and modes of IR region expansion, inversions and boundary shifts. During the analysis of the available data, we noticed some confusion in the assembly results, particularly regarding the orientation of the SSC region, which appears to be random. Of concern is the fact that only *Chieniodendron hainanense* exhibits inversion of the entire IR region. To clarify these issues, more plastomes of Annonaceae species need to be obtained for further investigation of their structural changes and evolutionary processes. This will provide additional information for the phylogenetics, genetics, and evolution of the Annonaceae.

## Data availability statement

The data presented in the study are deposited in the NCBI repository (https://www.ncbi.nlm.nih.gov/nuccore/), accession number: MN241494, MN241495, KU563738, MT742547, MN241491, MK087989, MT742546, MN241496, OQ687053, MN241488, MN241489, MH992130, MN241490, MW136266, MW829282, MZ936420, OK216144, MN253543, OQ682535, MN253544, OL979152, OM047203, OM914484, OM937139, MW018366, MK035708, MN016933, MK087990, NC_037005, NC_030504.

## Author contributions

JP: Data curation, Methodology, Writing – original draft. JH: Data curation, Methodology, Writing – original draft. TW: Writing – review & editing. YS: Writing – review & editing.
